# Submental Intubation

**DOI:** 10.1097/GOX.0000000000001896

**Published:** 2018-09-06

**Authors:** Edward A. Luce, Sonia M. Alvarez

**Affiliations:** From the Department of Plastic Surgery, University of Tennessee Health Science Center, Memphis, Tenn.

## Abstract

Acquisition of a secure airway is an essential element of the operative management of maxillofacial trauma. Of the options available, submental intubation is an alternative to tracheostomy. The access should be accomplished via a midline approach rather than lateral through the mylohyoid, an armored endotracheal tube utilized to prevent kinking, and the passage facilitated by use of wound dilators obtained from a percutaneous tracheostomy set.

## INTRODUCTION

The first description of a submental intubation was Altemir^1^ in 1986. Since that date, multiple publications have attested to the efficacy and low morbidity.^2–5^ Yet, several modifications have been adopted by us to facilitate the procedure.

Integral to the successful and safe care of the patient with complex maxillofacial trauma is the acquisition and maintenance of a secure airway. Contemporary trauma care may dictate in many patients, particularly if associated truncal and/or head injury is present, initial endotracheal intubation. At the time of the operative repair of the maxillofacial fractures, several options are available, the selection of the appropriate 1 hinges on the particular fracture, the status of the dentition, and individual patient considerations. Some examples include the necessity of establishment of intermaxillary fixation initially, the presence of craniofacial fractures such as a LeFort III with a cerebrospinal fluid leak, and a large maxillary tuberosity that creates limited retromolar space. The conventional options include peroral endotracheal intubation with placement of the endotracheal (ET) tube into the retromolar region or through space created by missing incisors, nasotracheal intubation placed by fiberoptic endoscopy and tracheostomy.

Certainly, the necessity to establish intermaxillary fixation (IMF) initially, nearly routine and complex in maxillofacial trauma, can narrow the choices of airway management. Retromolar tube placement can be difficult, particularly in the presence of erupted third molars or an enlarged maxillary tuberosity, and the tube can interfere with intraoral approaches. Nasotracheal intubation may not be advisable in the presence of a LeFort III fracture with or without CSF leak, given the concerns centered on unintended routing of the ET tube into the intracranial space via a skull base fracture. In other instances, as nasoethmoidorbital, nasoethmoidorbital (NEO) fractures, the necessity of transnasal intercanthal wiring and the placement of a cantilevered bone graft for a depressed nasal dorsum may preclude the nasal route for airway. A tracheostomy fulfills the criteria of a more remote access and a stable and secure airway, but at the expense of bypass of the protective mechanisms of the upper aerodigestive tract and the potential for direct contamination of the tracheo-bronchial tree. Other complications of tracheostomy include accidental dislodgment during or after the operative procedure, injury to the posteriorly located esophagus, major vascular injury, and pneumothorax.^6^

Although the submental route for airway access was initially described 30 years ago, the technique still has application for a certain component of the spectrum of craniofacial trauma. This report describes our procedure including some technical modifications and caveats.

## METHODS

**Fig. 1.**
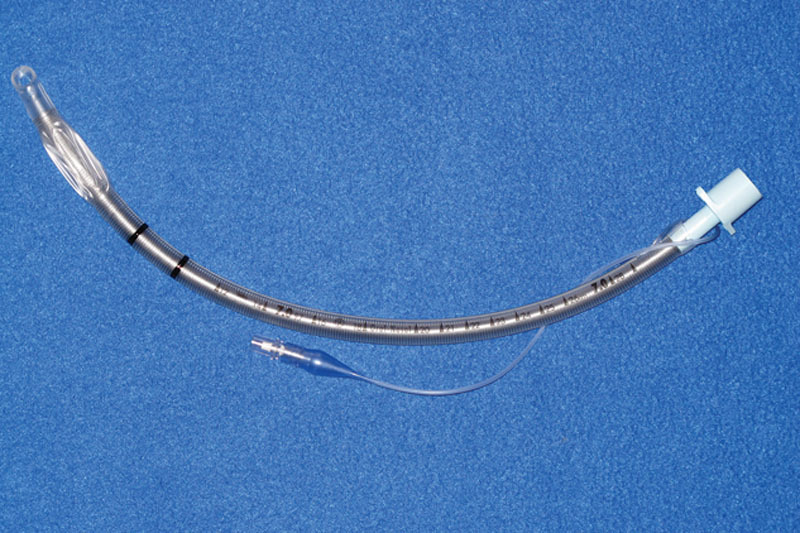
Armored endotracheal tube (Coviden Inc – Dublin, Ireland).

**Fig. 2.**
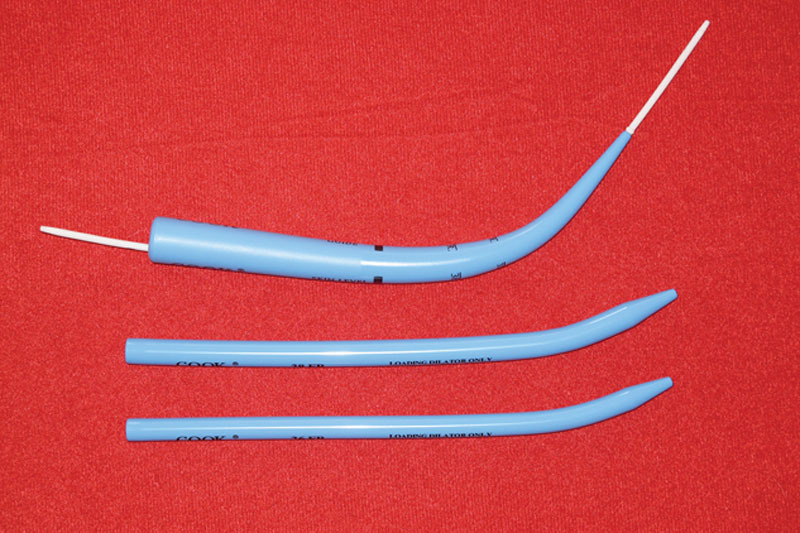
Percutaneous tracheotomy dilators (Cook Medical – Bloomington, Ind.).

**Fig. 3.**
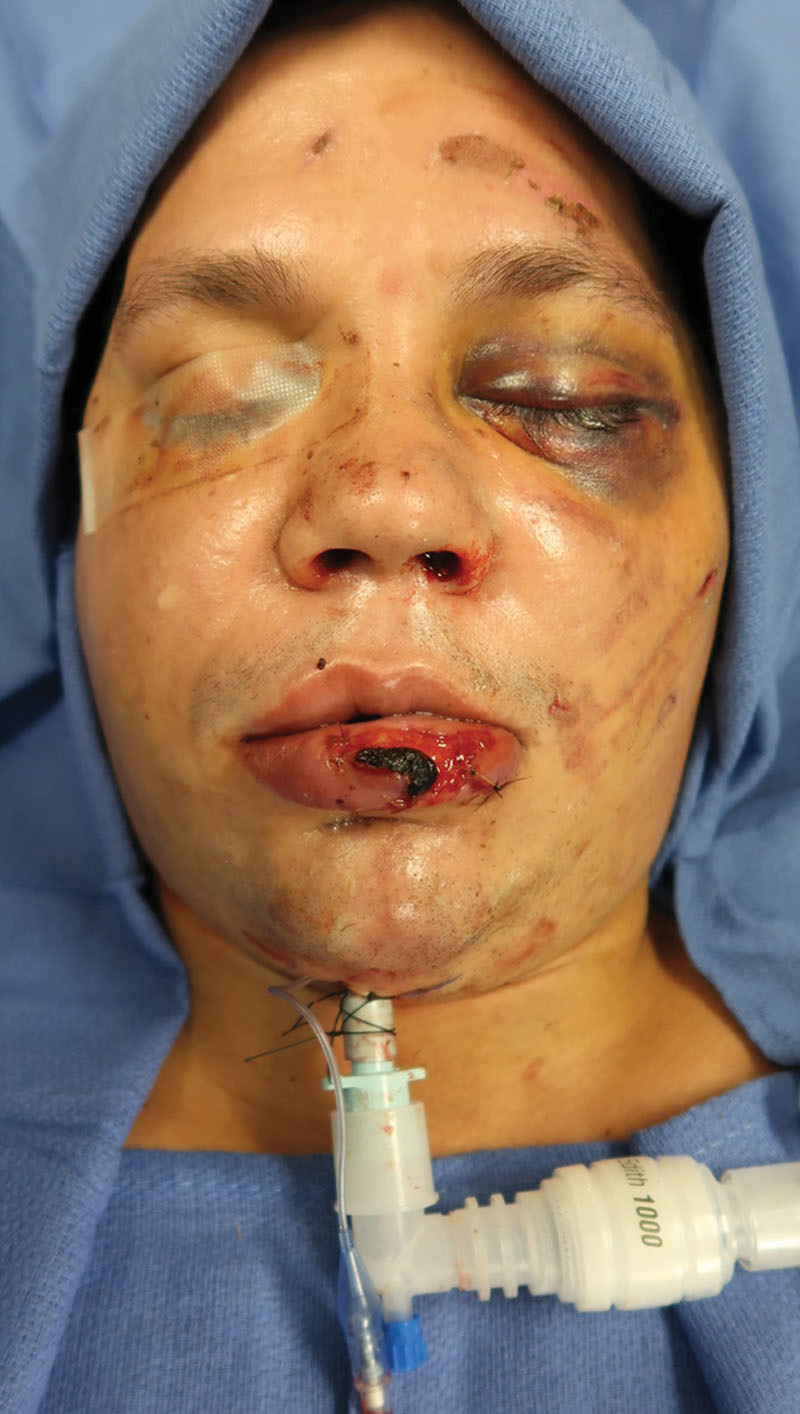
Submental intubation completed.

The patient is initially intubated orally with a wire-reinforced endotracheal tube (Fig. 1) (Coviden Inc – Dublin, Ireland) or if previously intubated with a standard ET tube, an intubation exchange is accomplished for the reinforced tube. The rationale for an armored tube is that a standard ET tube is prone to kinking at the exit point into the submental space. After intubation, a 2-cm transverse incision is placed within the submental crease in the midline, and dissection proceeds bluntly in the space between the paired anterior digastric muscles. Once the anterior floor of mouth is encountered, local vasoconstriction is administered and the mucosa incised over the projecting tip of a hemostat or scissors. At this juncture, the dilators from the percutaneous tracheostomy set (Fig. 2) (Cook Medical – Bloomington, Ind.) are utilized in progressively larger calibers until both the wound and submental route are of sufficient size to allow passage of the armored ET tube with ease. The anesthesia apparatus is uncoupled and the ET tube connector removed temporarily to facilitate passage of the tube alone. Assurance that the connector can be quickly and readily removed should be accomplished preoperatively, particularly if the tube has been previously resterilized. The largest dilator is then passed from percutaneous to peroral, connected to the ET tube, and drawn through the wound and secured after reconnection (Fig. 3).

Extubation can be performed in the operating room (OR) at the completion of the operation or if the individual situation so dictates at some point in the early postoperative period.

## RESULTS

Four patients, all with complex midface fractures, 2 with associated fractures of the mandible, were managed with submental intubation. Extubation was performed in the OR at the completion of the ORIF in 1 patient and 3 on the first postoperative day. All the patients healed uneventfully without prolonged drainage or salivary fistula.

## CASE DESCRIPTION

The patient was a 30-year-old law student who sustained LeFort III, NEO, and mandibular fractures in a motor vehicle accident and a lower lip laceration. Right nostril drainage was consistent with CSF rhinorrhea, but the patient was alert, oriented, and without injuries other than a foot fracture and ruptured right tympanic membrane. The patient’s facial fracture repair was accomplished on the third day postinjury.

The patient was induced by endotracheal intubation and a subsequent conversion to a submental location through an incision placed transversely in the submental crease, accomplished under sedation and facilitated by dilators obtained from a percutaneous tracheostomy set. Once placed in intermaxillary fixation, IMF, the fractures were managed with ORIF. At the completion of the procedure, the IMF was released and the patient was extubated. Previously placed sutures in the skin incision were secured.

**Fig. 4.**
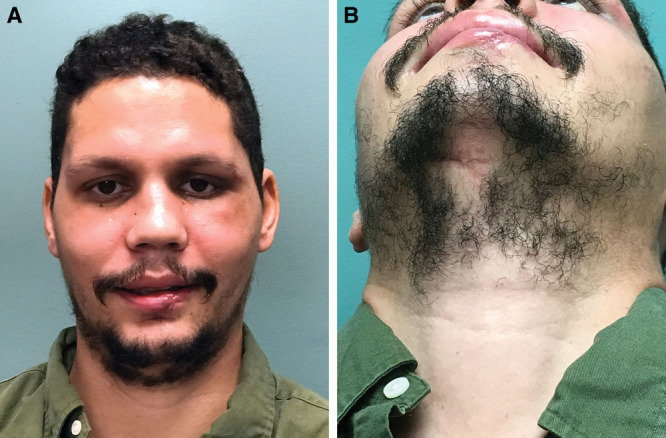
Patient is displayed at 6 weeks postoperative (A, B).

The postoperative course was uneventful as was healing of the submental incision (Fig. 4).

## DISCUSSION

A central component of the preoperative planning for repair of complex maxillofacial trauma is the airway. In most instances, either IMF is not required or the airway can be accessed by the nasotracheal or the retromolar route. Occasionally, circumstances dictate otherwise and the necessity of a tracheostomy looms as the option for the perioperative airway. For the head-injured patient or other concomitant injuries, ventilatory support may be necessary for an indeterminate period of time postoperatively, and a tracheostomy is appropriate and justified. For the patient whose injuries are confined to the maxillofacial region, tracheostomy is an additional insult and not without complications. Those complications consist of injury to the neighboring vascular and upper digestive tract structures, intra or immediate postoperative dislodgment and an unsightly scar.^6^ Submental intubation is a potential solution to this conundrum if executed properly.

The first consideration is the specific route of access. The most common description is that of a paramedian incision with blunt dissection through the mylohyoid.^4^ The structures at risk via this approach are the lingual nerve and the submandibular duct. An alternate passage is midline through a submental incision as utilized in this case series. The advantage is a more direct route with less dissection and a more concealed incision. The orifices of Wharton’s duct are potentially at risk but apparently easily recanalized as in the instance of excision of a submandibular duct stone. A vertical or sagittal mucosal incision between the sublingual gland orifices can also minimize injury. The second consideration is the endotracheal tube utilized. Because the standard ET tube is subject to kinking at the point of exit submentally, a wire-reinforced or armored tube as utilized in laryngectomies is preferable. If the armored ET tube is reusable and autoclaved in the past, assurance must be established that the connector is easily removed before intubation since removal of the connector after intubation facilitates passage from intra- to extra oral through the submental incision.

The third consideration is the use of dilators to enlarge the submental pathway. Solid plastic dilators of a variety of caliber size are available on a percutaneous tracheostomy set. The dilators are modified at 1 end to enable connection to a tracheostomy tube, or in this instance, an endotracheal tube. Once the passage appears sufficient, the ET tube is disconnected from the anesthesia apparatus, the ET tube connector removed, the dilator passed from the neck and connected to the ET tube, and subsequently passed extra orally and reconnected to the anesthesia apparatus. Of course, the tube should be secured with a stout suture at this point. The skin wound can be sutured also to provide a more rapidly healed submental incision. Dependent on individual patient circumstances, extubation can be accomplished immediately postoperative or in the early postoperative period.

Contraindications to submental intubation include the necessity for prolonged assisted ventilation as, for example, a severely head-injured patient, although an option would be reentry of the endotracheal tube into the oral cavity. Certainly, the presence of significant lacerations in the anterior oral cavity would be a relative contraindication as well.

## CONCLUSIONS

Intubation by the submental route offers a safe and facile alternative to tracheostomy for airway access in the patient with complex midface fractures. Our experience affords several technical suggestions including specific incision and space location, type of endotracheal tube, and use of dilators for passage of the tube.
